# Group size effects on inter-blink interval as an indicator of antipredator vigilance in wild baboons

**DOI:** 10.1038/s41598-018-28174-7

**Published:** 2018-07-03

**Authors:** Akiko Matsumoto-Oda, Kohei Okamoto, Kenta Takahashi, Hideki Ohira

**Affiliations:** 1 Graduate School of Tourism Sciences, University of the Ryukyus, Okinawa, Japan; 2 Mpala Research Centre, Nanyuki, Kenya; 3 Graduate School of Informatics, Nagoya University, Nagoya, Japan; 4 Faculty of Science, University of the Ryukyus, Okinawa, Japan; 5 Win-K Corporation, Tokyo, Japan

## Abstract

Vigilance in animals is an important means for predator detection. Animals living in groups reduce their predation risk as more individuals are present. In contrast to most other animals studied, many studies on primates do not support the prediction that individual vigilance will decline as group size increases. For animals to obtain visual information during vigilance behaviour, their eyes must be open. Therefore, if animals are able to perceive differential risk of predation, the inter-blink interval (eye-opening) should increase, and the blink duration (eye-closure) should decrease under higher predation risk. We tested this prediction by measuring inter-blink interval in wild anubis baboons (*Papio anubis*) in peripheral and centre individuals within a group, and between larger and smaller groups. We found that the inter-blink interval for young males, often located at the front edge of the group, was longer than that of adult males, adult females, and young females, often located in the center of the group, and that the inter-blink interval for adult males was longer when the group was smaller. These results suggest that inter-blink interval can be used as an indicator of primate vigilance toward predators.

## Introduction

Predation is one of the most important selective pressures for animal behaviours^1^ and group-living is widespread among most taxa of the animal kingdom including mammals, birds, fishes and insects^2^. Therefore, the relationship between benefits of anti-predation and group-living is a central issue in behavioural ecology. It has been well documented that forming a group provides anti-predatory advantages^3–7^. This group size effect on vigilance is often explained by the group-vigilance hypothesis^7^ (also called the “many-eyes effect”^8^ or “collective detection”^9^) and risk-dilution hypothesis^4^.

The group-vigilance hypothesis predicts that the gregariousness of individual animals has a cumulative effect on vigilance and increases the possibility of predator detection earlier. Whether predators succeed in hunting depends on whether surprise attacks succeed^5,7^, and prey animals must constantly monitor potential threats. Vigilance has been defined as an animal visually scanning the surrounding environment and is often thought of as a behaviour targeting predators^10^. In addition, individual animals belonging to a large group do not have to be vigilant for the same amount of time as when they live in a small group. Such individual animals also have advantage that they can distribute more of their time to other activities, such as foraging, without reducing the total amount of group vigilance, depending on their group’s members^7,11–13^. A decrease of individual vigilance efforts associated with an increase in group size is widely reported in mammals and birds^14,15^.

Many studies on non-human primates, however, have not supported the group-vigilance hypothesis^16^. In the studies of non-human primates until 2000, 9 of 11 studies showed no group size effect^16^. The latest review on the group size effect between vigilance and group (including subgroup) in non-human primates reported that 6 of 15 studies had no effect, 3 showed a positive effect and 6 showed a negative effect^17^. In addition, one recent study reported that the group sizes in vervet monkeys (*Chlorocebus pygerythrus*) did not show any substantial relationship with the access/encounter rate with leopards^18^. The above two reviews pointed out the possibility that the diversity of results in primates might come from fundamental differences in the method adopted^16,17^. Due to body structure, many vertebrates must bring their mouths close to food during feeding, meaning that vigilance during feeding is difficult. Therefore, vigilance is marked by an individual lifting its head away from the ground and paying attention to its surroundings, either with or without a scan of environment, in studies of artidactyls and birds^19^. Contrastingly, primates can convey food to their mouths using their hands, and head lifting is a poor marker of measuring vigilance. Hence, previous studies on primates used various definitions of vigilance behaviours, such as the frequency of the focal animal’s head movement^20^, angle of the animal’s head^21^, the duration or frequency of the scan^22^, or a line of sight outside the reach of arm^23^ and combinations thereof ^24^. Previous results in primates were based on different methods and cannot be compared collectively. This suggests it is necessary to develop indicators of vigilance behaviours that can be used on both primates and other mammals.

Blinking and the inter-blink interval is a possible indicator of vigilance for predators. For animals to obtain visual information during vigilance behaviour, their eyes must be open. Although blinking is necessary to prevent drying of the eyes and to protect the eyes from foreign body damage^25^, animals are not able to access visual information while blinking because the eyelids temporarily block visual information. Blinking behaviour has been studied in humans but has not been extensively studied in other animals. One study of nonhuman primates^26^ and two studies of birds^27,28^ examined the relationship between blinking and potential danger. The study of non-human primates in zoos compared the blink rate among 71 species, showed that the blink rate was not affect by habitat types (arboreal, semiarboreal, terrestrial) but increased as average group size increased^26^. Although the study was concluded that the blink rates acquired a role in social communication, inter-species examination on blink rate and group size still remains. Wild American crows (*Corvus brachyrhynchos*) reduced blink rates to fix their gaze on a possible danger^27^. Adult male peacocks (*Pavo cristatus*) shortened blinking after a predator was revealed^28^. These studies suggest that animals suppress blinking in alert situations and acquire more information by opening their eyes for a longer time. Collectively, to minimize loss of information, blinks need to be strategically adjusted.

The blink rate depends on the situation animals are placed in. The default mode network of the brain adjusts blinking timing in both non-human primates^29^ and humans^30^. Non-human primates blink half as much as humans, with 71 species of non-human primates blinking an average of 10.9 times per minute^26^. In gorillas (*Gorilla gorilla gorilla*), which blink more frequently than other non-human primates, the duration of blinking is 335 ms and blinking occurs 29.4 times/minute^26^. Therefore, it can be calculated that the eyes are closed 16.4% of the time. In humans, blinking changes with cognitive tasks demanding attention and concentration. Blinking tended to increase as time spent on tasks increased^31^ and decreased during engagement in difficult tasks^32^. Most wild primates inhabit environments with high predation risk. Detecting a predator is a difficult task, and if the predation risk is high, primates are likely to suppress the number and duration of blinks and to extend the duration of open-eye periods.

**Figure 1:**
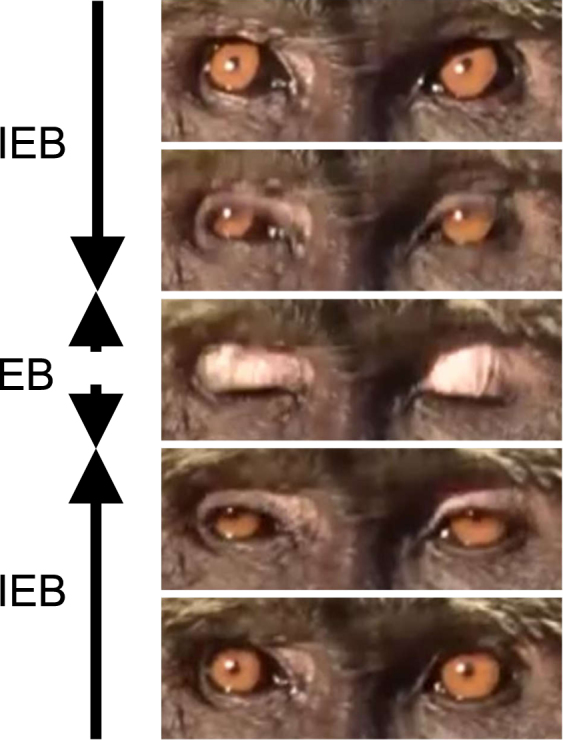
Definition of inter-blink interval (IBI) and blink duration (BD). Blink duration was defined as a period during which the eyelids hid the pupil^37^. Inter-blink interval was defined as time except for blink duration. Blink duration and inter-blink interval were measured by the Movie maker in 1/100 second.

In this study, we tested two predictions in a group of wild anubis baboons (*Papio anubis*): (1) between peripheral and centre individuals and (2) between larger and smaller groups. Assuming that a predator chooses the nearest individual as prey, the degree of risk varies depending on a spatial position in a group, for example, whether an individual is at the periphery or at the centre of a group. Because individuals in the periphery are more at risk of predation than individuals in the centre^33–35^, individuals in the periphery are expected to have an extended duration of open-eye periods and a decrease the number and duration of blinks. Also, according to the group vigilance hypothesis, it is predicted that individuals belonging to a small group are riskier than individuals of a large group and are expected to have increased vigilance. Six species of large carnivores live in the same habitat with the subject baboon group^36^. We recorded videos of adult males in 2013, and adult males, young males, adult females and young females in 2016 (Table 1). To measure open-eye periods, we quantified blink duration and inter-blink interval, defined as the period during which the eyelids hid the pupil^37^ and that during which the eyelids did not hide the pupil, respectively (Fig. 1). The group size of baboons decreased from 72 in 2013 to 53 in 2016 because of ordinary males’ transfer and disappearance of infants and old individuals.

**Table 1:** Summary of the number of individuals, inter-blink interval (IBI) and blink duration (BD).

Period (group size)	Sex∙age^*1^	Median of IBI range (0.01 sec)	Median of BD range (0.01 sec)	The number of individuals in AI group	The number of individuals analysed	The number of IBI	The number of BD	The number of IBI per individual (Weighted average ± SD)	The number of BD per individual (Weighted average ± SD)
Sep-Oct, 2013 (72 heads)	♂ ∙ Adult	113.00 4.00–1380.00	13.00 3.00–397.00	12	9	193	229	213.68 ± 66.79	22.69 ± 7.58
Sep-Oct, 2016 (53 heads)	♂ ∙ Adult	220.00 3.00–5154.00	13.00 3.00–278.00	4	4	418	448	464.07 ± 66.81	21.68 ± 8.72
	♂ ∙ Young	373.00 3.00–5906.00	13.00 3.00–316.00	9	8	363	390	674.91 ± 233.52	21.68 ± 8.72
	♀ ∙ Adult	151.50 7.00–3634.00	13.00 3.00–213.00	13	6	92	100	270.08 ± 56.90	16.10 ± 4.19
	♀ ∙ Young	183.00 6.00–3137.00	14.00 3.00–286.00	3	2	111	116	357.81 ± 15.05	19.13 ± 3.5

*1: The age classes for each sex are defined according to the previous studies of anubis baboons. Males’ age-classes were judged based on apparent weight, the sizes of canines and testicles, and immigrational history^56,57^. ‘Adult males’ are defined as over 8 years old when completely erupted and are reproductive mature. ‘Young males’ are 4–8 years old. Females are considered ‘adults’ after their first pregnancy, and ‘young’ after the first sexual swelling experience. The first birth by females averaged at 6.9 years old, and the first sexual swelling averaged 4.8 years old^58^.

## Results

### Age-sex classes, and the duration of inter-blink and blink periods

**Figure 2:**
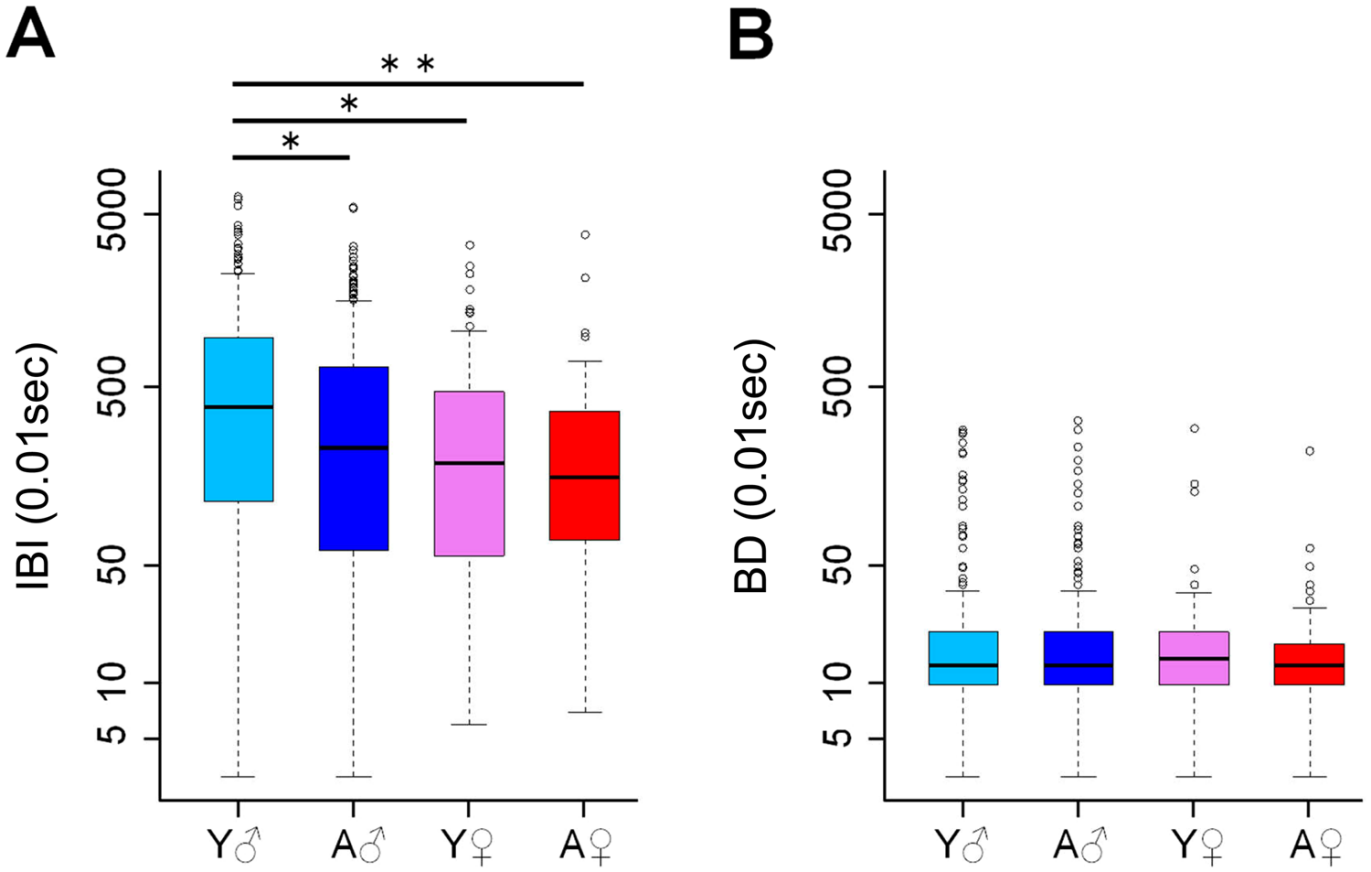
Age and sex classes and the duration of inter-blink interval (IBI) and blink duration (BD) periods. (**A**) Young males showed longer inter-blink interval than those of other three classes. (**B**) There was no difference in blinking duration between four age-sex classes. Thick horizontal lines show the medians, boxes show the inter-quartile range (IQR), and whiskers show 1.5 times the IQR. Abbreviations: Y♂, Young male; A ♂, Adult male; Y♀, Young female; A♀, Adult female. *p < 0.05 and **p < 0.01, tested by lsmeans using “lsemans” package.

In 2016, the inter-blink interval of males was longer than that of females (Fig. 2). This difference in sex was significant (ART-ANOVA, F(1, 16) = 12.37, p = 0.003). Moreover, the inter-blink interval of young individuals was significantly longer than that of old individuals (age: F(1, 16) = 6.77, p = 0.019). There was no significant fixed effect of age and interaction effect between age and sex (F(1, 16) = 0.97, p = 0.339). Post hoc analyses indicated that young males opened their eyes for longer periods than other age/sex classes (contrast test, young male vs. adult male: mean difference ± se = −88.83 ± 24.87, p = 0.012; young male vs. adult female: −158.52 ± 40.47, p = 0.001; young male vs. young female: −126.03 ± 37.60, p = 0.019). No significant difference was found among the other age/sex classes (adult male vs. adult female: −69.69 ± 39.93, p = 0.334; adult male vs. young female: −37.20 ± 37.02, p = 0.749; adult female vs. young female: −32.49 ± 48.89, p = 0.909).

Young males tended to be in front of the study group with respect to the direction of travel. For example, when the group crossed a river, the first 20% included more young males (mean ± sd = 1.88 ± 1.15) than expected (1.19 ± 0.25) based on their representation in the group (Exact Wilcoxon signed-rank test, n = 24, V = 65.6, p = 0.014).

There were no significant differences in the blink duration between age classes (F(1, 16) = 1.03, p = 0.325) or between sexes (F(1, 16) < 0.01, p = 0.942), and no significant interaction among these variables (F(1, 16) = 0.55, p = 0.467) (Fig. 2).

### Group size, and the duration of inter-blink and blink periods

**Figure 3:**
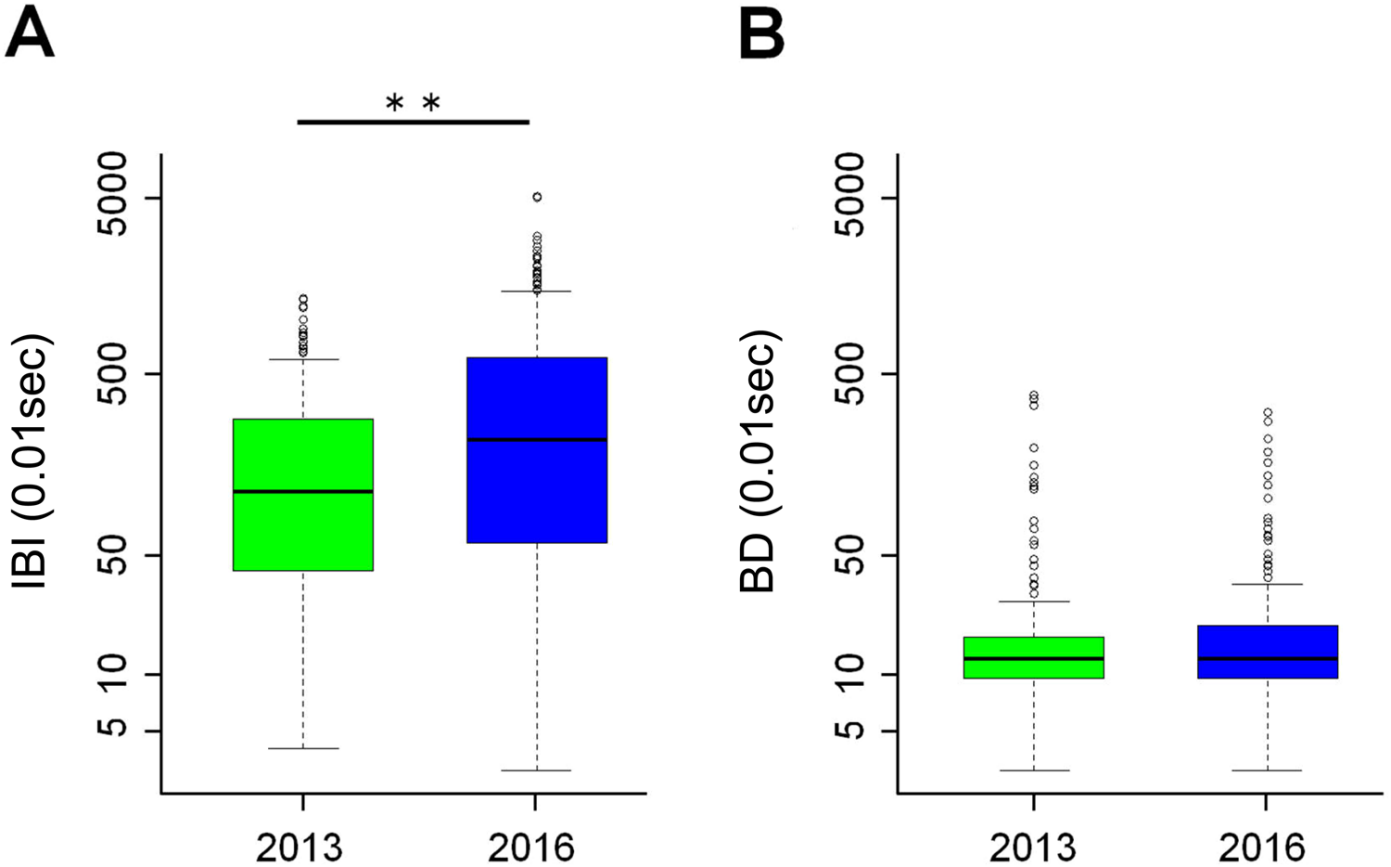
Group size, and the inter-blink interval (IBI) and blink duration (BD). The size of a wild anubis baboon group, AI, was larger in 2013 than in 2016. (**A**) Adult males in 2016 showed longer inter-blink interval than that of in 2013. (**B**) There was no difference in blinking duration between adult males in 2013 and in 2016. Thick horizontal lines show the medians, boxes show the inter-quartile range (IQR), and whiskers show 1.5 times the IQR. **p < , tested by lsmeans using “lsemans” package.

**Figure 4:**
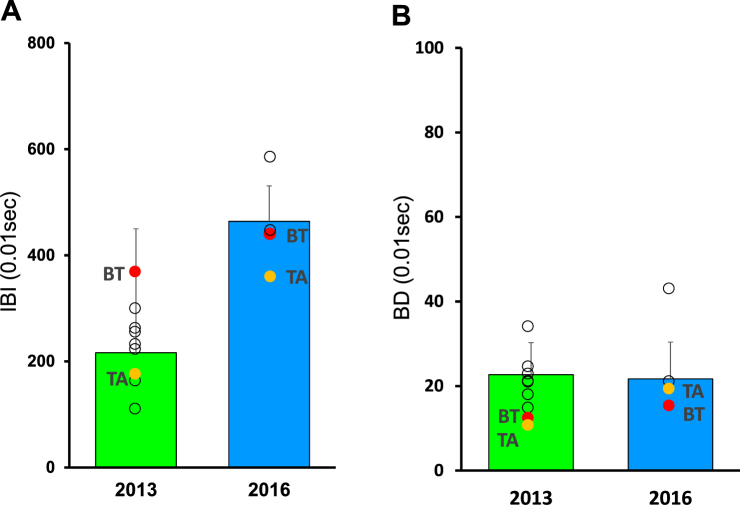
Group size, and the inter-blink interval (IBI) and blink duration (BD) of individual adult males. (**A**) Weighted average value of the inter-blink interval of 9 adult males in 2013 and of 4 adult males in 2016. (**B**) Weighted average value of the blink duration of 9 adult males in 2013 and of 4 adult males in 2016. The whiskers show standard deviation of the weighted average value of adult males. The white circles indicate weighted average values of individual males. Two males, BT and TA, belonged to the AI group in both 2013 and 2016. The red and yellow circles indicate the weighted average values of BT and TA, respectively.

The group size was smaller in 2016. Inter-blink interval of adult males was significantly longer in 2016 (median = 220.00) compared to 2013 (median = 117.00) (F(1, 8) = 28.45, p < 0.001) (Fig. 3). In both years of 2013 and 2016, the AI group contained the same two adult males, BT and TA. Focusing on the inter-blink interval of individual adult males, the inter-blink interval of both BT and TA were found to be longer in 2016 from 2013 (Fig. 4).

The blink duration did not differ between 2013 and 2016 (F(1, 8) = 0.21, p = 0.65) (Fig. 3). The blink duration of BT and TA did not seem to be different between 2013 and 2016 (Fig. 4).

## Discussion

**Figure 5:**
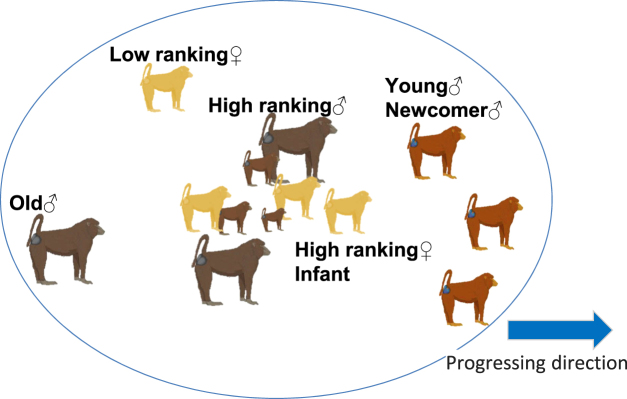
Conceptual diagram of spatial position in a group of baboons. There were tendencies that mothers and infants were at the center of the group^39,40^. Males were at the periphery than females, and young males tended to be forward in the direction of travel and older males were at the most peripheral position^41–43^.

The inter-blink interval of young males was longer than those of other age-sex classes in the AI group. Two factors may explain the longer inter-blink interval observed among young males. The first is vigilance for predation. It is likely that the relatively dangerous spatial position of young males within the group increased vigilance for predation^4,38^. Studies on the spatial position of primates are usually difficult because animals move in three dimensions. However, to summarize some previous studies on spatial position in yellow baboons (*P*. *cynocephalus*) and chacma baboons (*P*. *ursinus*), females with infants tended to locate at the middle of group^39,40^, high-ranking adult males were with the nursing females, young males were at the front of travel direction^41,42^ and old males fell behind^43^ (Fig. 5). In this study also, young males tended to be at front of the direction of the group. Individuals at the edge of a group may be at greater risk of predation than those in the middle. Buss^39^ suggested that lionesses at Moremi, Botswana, may anticipate the movement of chacma baboon groups and wait in ambush for baboons to approach because baboons were attacked seven times from the front of the group and one time from behind. In that case, young males are more likely to be chosen as prey. Therefore, they must be more vigilant than individuals located in the middle place. Furthermore, it is reasonable for young males to be more vigilant if predators may be less likely to target vigilant individuals^44,45^.

The second possible factor of the longer inter-blink duration in young males is vigilance for attacks from other group members. However, indirect evidence from other studies suggests that this possibility is less likely. In anubis baboons at Gombe in Tanzania^46^, males were injured more than females and males over 9 years old were the most often injured. Similarly, in yellow baboons, adult males were injured in attacks more often than subadult males^47^. Since we found that inter-blink duration was increased only among young males, it seems unlikely that vigilance is intended to protect against attacks within the group. However, since the basis for this conclusion is still insufficient, the relationship between age and inter-blink duration will be elucidated in the future.

The inter-blink interval of adult males in the small group was longer than that in the large group, which supports the group vigilance hypothesis. Previous studies in non-human primates, that showed the negative effects between group size and vigilance, compared the vigilance rates among different groups^48–52^. Moreover, these studies compared the group size based on the number of females because the groups included only 1 or 2 males^49–51^ or no male^52^. Therefore, this is the first study that analysed the relationship between population size/number of males and vigilance duration in the same group. However, in our study, group size and number of adult males both decreased, so we could not independently assess these two variables. In one study, among four groups of *Cebus capucinus*, there was a negative correlation between the number of males in the group and average vigilance rate, but the overall average vigilance rate of each group was not related to group size^53^. Future studies should clarify whether vigilance depends on group size or number of males by comparing inter-blink interval for adult males and other age/sex classes. Also, it may be necessary to compare inter-blink interval in the same individuals included in different sized groups. Although there were only 2 adult males belonging to both groups of the two years and the data size was small in this study, it is notable that the inter-blink interval of the two adult males showed the same tendency of becoming longer as group size decreased in 2016. There is room for further investigation whether inter-blink interval can be adopted as an indicator of vigilance in other species.

We found that blink duration was insensitive to age, sex or group size. On the one hand, blinking is necessary to prevent the cornea from drying out, and to evenly distribute tears. On the other hand, visual information is blocked during blinking, meaning that danger may not be detected. The optimal blink duration thus might be determined by a trade-off between physiological needs and predator detection. In the wild, delayed predator detection may be costly to survival; therefore, natural selection is expected to favour short blink duration. The predation pressure suffered by the baboons of AI group corresponded to three individuals being killed in a group of 50 individuals every year, and the median blink duration of the baboons in this habitat was 0.13 seconds. This value is shorter than the average blink duration in 71 species of non-human primates in zoos (0.21 seconds)^26^ and in modern humans (0.3 seconds), who are presumed to have very low predation pressure. Such a short blink duration would be more effective to quickly detect predators and escape. For example, mallard ducks, which keep one eye open during sleep, initiate escape behaviour within only 0.17 seconds of exposure to videos of attacking predators^54,55^. This value was close to the observed blink duration in the AI baboon population.

Finally, as this study shows, non-human primate studies that employed head turning as a marker of vigilance behaviour have not found any correlation between group size and vigilance behaviour, as have studies in other animals. However, utilizing the duration of open-eye time as a marker of vigilance behaviour, we identified a group size effect on vigilance behaviour in anubis baboons. To clarify the causal relationship between the duration of eye opening (and the duration of blinking) and the detection of danger, as has been studied in humans, it is necessary to know the relationship between blinking and animal concentration and the relationship between blinking and incorporation of new information into the brain. Further studies of blinking function on non-human primates and other mammals are necessary.

## Methods

### Study site and subjects

The analyses focused on wild anubis baboons, the AI group, in Mpala on the Laikipia Plateau of central Kenya (0.17°N, 36.53°E). The AI group has been habituated since 2011 by AMO. We conducted two periods of field research on the AI group; September-October 2013 and August-September 2016.

Anubis baboons live in multi-male/multi-female social groups, and males transfer between groups after reaching sexual maturity. ‘Adult males’ are defined as over 8 years old and completely grown and reproductive mature^56,57^. ‘Young males (or adolescents)’ are 4–8 years old. The ages of transferred males were unknown, and the ages of natal males were also unknown except for individuals who were born after the habituation started. Therefore, males’ age-classes were judged based on apparent weight, sizes of canines and testicles and immigrational history. Females are considered ‘adults’ after their first birth, and ‘young (or adolescent)’ after the first sexual swelling experience. The first birth by females was around at 6–7 years old, and the first sexual swelling was around at 4–5 years old^58^. Females’ age-classes were also judged based on apparent body size, nipple length and size of sexual swelling.

The group size of the AI in 2013 was larger than that in 2016 (Table 1). The number of adult males also decreased from 11 to 4 during this time and 2 adult males were in the group in both years. The decrease in the size of the AI group was brought about by the usual individual disappearance (mainly males transfer and disappearance of old individuals). The predation rate was calculated as the sum of following three items: (a) number of owners of carcasses or remains; (b) number of individuals disappearing during the night and direct predator information (voice or observation) in the morning and evening and indirect predator information (GPS location of leopard and baboon) and (c) number of healthy females and immature individuals disappearing during the day time. Predation rate on the AI group during 2011–2016 was estimated at 0.06 individuals/year.

The focal targets were adult males in 2013, and adult males, young males and adult females in 2016. Videos of the individual faces were recorded with a Sony HDW-750 camera (30 fps, 21.1 megapixels) or a Sony DSC-HX 60 V (60 fps, 21.1 megapixels) camera. Both the study periods were within the short dry season, and we took video clips near their sleeping sites on morning of sunny days. The focal targets sat and watch the environment, and they were not engaged in other activities, such as feeding or self-grooming. Although previous zoo studies of blink duration in primates have used videos of more than 5 minutes duration^26^, it was impossible in the wild to take videos of faces for more than 5 minutes because animals moved or changed head angle. Therefore, we unrestricted the duration of the video clips.

Blink duration was defined as the time that the eyelid completely concealed the pupil (Fig. 1). The inter-blink interval was defined as time other than the blink duration. The inter-blink interval and the blink duration were analysed in 0.01-second segments using Movie Maker software. Blinking tend to link with head movement^26^. Accordingly, blinking with head movement was not included in our data because the moment of blinking cannot be confirmed on a video clip even with a slight head angle change. Moreover, blinking with yawning were also excluded from the analyses. Inter-blink interval and blink duration were measured on computers twice by two persons.

To understand an animal’s spatial position in the group, we recorded the animal’s order of travel as they crossed the river. In operation, we defined the first 20% of animals as animals in the forward position.

### Ethics Committee

The field research in Kenya was approval from the Kenya governmental agencies National Council for Science and Technology (permit No. NCST/PRI/12/1/BS/240), National Commission for Science, Technology & Innovation (NACOSTI/P16/84320/12475), and Kenya Wildlife Service (KWS/BRM/5001).

### Statistical analyses

**Figure 6:**
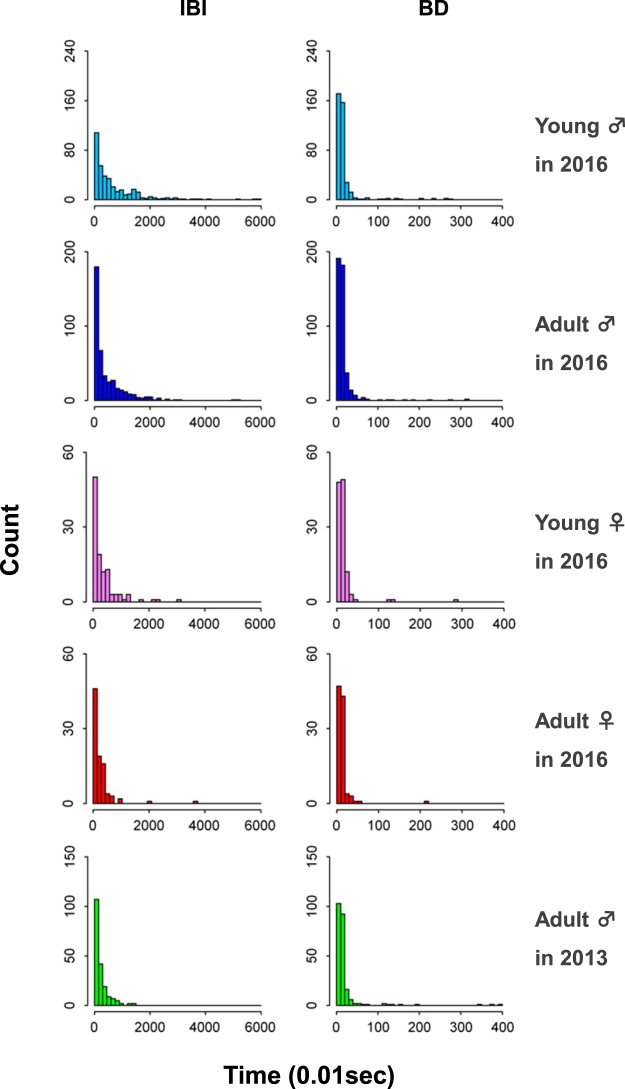
Distribution of inter-blink interval (IBI) and blink duration (BD). Left column: Histograms showing the distribution of inter-blink interval. Right column: Histograms showing the distribution of blink duration. The top 4 rows represent the data of 4 age-sex classes in 2016 and the bottom row represents the data of adult male in 2013. See also Table 1.

All analyses were performed using R software (version 3. 3. 3)^59^. As the data were not normally distributed (Fig. 6), we employed a repeated measures ANOVA of aligned rank transformed data (ART-ANOVA) with individual as an error term, using the “ARTool” package^60^ and conducted a post hoc pairwise comparison using the “lsmeans” package^61^ with the Tukey’s test for multiple comparisons. We performed an exact Wilcoxon matched-pairs signed-rank test to compare expected and actual numbers of young males in the front of the group during the river crossing using the “exactRankTests” package in R.

### Data availability

The datasets generated during and/or analysed during the current study are available from the corresponding author on request.

## References

[1] Krebs, J. R. & Davies, N. B. An Introduction to Behavioural Ecology. 3rd edition. (Blackwell Scientific Publications, 1993).

[2] Pulliam, H. R. & Caraco, T. Living in groups: Is there an optimal group size? in Behavioural Ecology: an Evolutionary Approach, 2nd edition (ed. Krebs, J. R. & Davies, N. B.) 122–147 (Blackwell Scientific Publications, (1984).

[3] De Vos A, O’Riain MJ (2010). Sharks shape the geometry of a selfish seal herd: experimental evidence fromseal decoys. Biol. Lett..

[4] Hamilton WD (1971). Geometry for the selfish herd. J. Theor. Biol..

[5] Krause, J. & Ruxton, G. D. Living in groups. (Oxford University Press, 2002).

[6] Milinski M (1984). A predator’s cost of overcoming the confusion effect of swarming prey. Anim. Behav..

[7] Pulliam HR (1973). On the advantages of flocking. J. Theor. Biol..

[8] Powell GVN (1974). Experimental analysis of the social value of flocking by starlings (Sturnus vulgaris) in relation to predation and foraging. Anim. Behav..

[9] Lima SL (1995). Back to the basics of anti-predatory vigilance: the group-size effect. Anim. Behav..

[10] Beauchamp, G. Animal vigilance: monitoring predators and competitors. (Academic Press, 2015).

[11] Bednekoff PA, Lima SL (1998). Randomness, chaos and confusion in the study of antipredator vigilance. Trends Ecol. Evol..

[12] Dehn MM (1990). Vigilance for predators: detection and dilution effects. Behav. Ecol. Sociobiol..

[13] Roberts G (1996). Why individual vigilance declines as group size increases. Anim. Behav..

[14] Elgar MA (1989). Predator vigilance and group size in mammals and birds: a critical review of the empirical evidence. Biol. Rev..

[15] Quenette PY (1990). Functions of vigilance behavior in mammals: a review. Acta Oecol. Int. J. Ecol..

[16] Treves A (2000). Theory and method in studies of vigilance and aggregation. Anim. Behav..

[17] Allan ATL, Hill RA (2018). What have we been looking at? A call for consistency in studies of primate vigilance. Am. J. Phys. Anthropol..

[18] Isbell LA, Bidner LR, Van Cleave EK, Matsumoto-Oda A, Crofoot MC (2018). GPS-identified vulnerabilities of savannah-woodland primates to leopard predation and their implications for early hominins. J. Hum. Evol..

[19] Walther FR (1969). Flight behaviour and avoidance of predators in Thomson’s gazelle (Gazella thomsoni Geunther 1884). Behaviour.

[20] Gaynor KM, Cords M (2012). Antipredator and social monitoring functions of vigilance behaviour in blue monkeys. Anim. Behav..

[21] Barros M, Alencar C, Silva MADS, Tomaz C (2008). Changes in experimental conditions alter antipredator vigilance and sequence predictability in captive marmosets. Behav. Processes.

[22] Nunes DM, Gonçalves I, Emile N, Barros M (2010). Bimodal temporal organization of specific vigilance behaviors in captive black tufted-ear marmosets (Callithrix penicillata). Behav. Process.

[23] Treves A (1998). The influence of group size and neighbors on vigilance in two species of arboreal monkeys. Behaviour.

[24] Alberts SC (1994). Vigilance in young baboons: effects of habitat, age, sex and maternal rank on glance rate. Anim. Behav..

[25] Nakamori K, Odawara M, Nakajima T, Mizutani T, Tsubota K (1997). Blinking is controlled primarily by ocular surface conditions. Am. J. Ophthalmol..

[26] Tada H, Omori Y, Hirokawa K, Ohira H, Tomonaga M (2013). Eye-blink behaviors in 71 species of primates. Plos One.

[27] Cross DJ (2013). Distinct neural circuits underlie assessment of a diversity of natural dangers by American crows. Proc. R. Soc. B..

[28] Yorzinski JL (2016). Eye blinking in an avian species is associated with gaze shifts. Sci. Reports.

[29] Guipponi O, Odouard S, Pinède S, Wardak C, Ben Hamed S (2015). fMRI cortical correlates of spontaneous eye blinks in the nonhuman primate. Cereb. Cortex.

[30] Nakano T, Kato M, Morito Y, Itoi S, Kitazawa S (2013). Blink-related momentary activation of the default mode network while viewing videos. Proc. Natl. Acad. Sci. USA.

[31] Fukuda K, Stern JA, Brown TB, Russo MB (2005). Cognition, blinks, eye-movements, and pupillary movements during performance of a running memory task. Aviat. Space Env. Med..

[32] Karson CN (1981). Speaking, thinking, and blinking. Psychiatry Res..

[33] Bednekoff PA, Ritter R (1994). Vigilance in Nxai Pan Springbok. Antidorcas marsupialis. Behaviour.

[34] Burger J, Gochfeld M (1994). Vigilance in African mammals: differences among mothers, other females, and males. Behaviour.

[35] Underwood R (1982). Vigilance behaviour in grazing African antelopes. Behaviour.

[36] Young TP, Okello BD, Kinyua D, Palmer TM (1997). KLEE: A long-term multi-species herbivore exclusion experiment in Laikipia, Kenya. Afr. J. Range Forage Sci..

[37] Maus GW (2017). Target displacements during eye blinks trigger automatic recalibration of gaze direction. Curr. Biol..

[38] van Schaik CP, van Noordwijk MA (1989). The special role of male Cebus monkeys in predation avoidance and its effect on group composition. Behav. Ecol. Sociobiol..

[39] Busse CD (1984). Spatial structure of chacma baboon groups. Int. J. Primatol..

[40] Cowlishaw G (1999). Ecological and social determinants of spacing behaviour in desert baboon groups. Behav. Ecol. Sociobiol..

[41] Collins DA (1984). Spatial pattern in a troop of yellow baboons (Papio cynocephalus) in Tanzania. Anim. Behav..

[42] Rhine RJ (1975). The order of movement of yellow baboons (Papio cynocephalus). Folia Primatol..

[43] Rhine RJ, Westlund BJ (1981). Adult male positioning in baboon progressions: order and chaos revisited. Folia Primatol..

[44] FitzGibbon CD (1989). A cost to individuals with reduced vigilance in groups of Thomson’s gazelles hunted by cheetahs. Anim. Behav..

[45] Lima SL (1994). On the personal benefit of anti-predatory vigilance. Anim. Behav..

[46] MacCormick HA (2012). Male and female aggression: lessons from sex, rank, age, and injury in olive baboons. Behav. Ecol..

[47] Drews C (1996). Contexts and patterns of injuries in free-ranging male baboons (Papio cynocephalus). Behaviour.

[48] Isbell LA, Young TP (1993). Social and ecological influences on activity budgets of vervet monkeys, and their implications for group living. Behav. Ecol. Sociobiol..

[49] de Ruiter JR (1986). The influence of group size on predator scanning and foraging behaviour of wedgecapped capuchin monkeys (Cebus olivaceus). Behaviour.

[50] Gosselin-Ildari AD, Koenig A (2012). The effects of group size and reproductive status on vigilance in captive Callithrix jacchus. Am. J. Primatol..

[51] Kazahari N, Agetsuma N (2010). Mechanisms determining relationships between feeding group size and foraging success in food patch use by Japanese macaques (Macaca fuscata). Behaviour.

[52] Hill, R. A. & Cowlishaw, G. Foraging female baboons exhibit similar patterns of antipredator vigilance across two populations. in Eat or be eaten: predator sensitive foraging among primates (ed. Miller, L. E.) 187–204 (Cambridge University Press, 2002).

[53] Rose LM, Fedigan LM (1995). Vigilance in white-faced capuchins, Cebus capucinus, in Costa Rica. Anim. Behav..

[54] Manoach DS, Stickgold R (2016). Sleep: keeping one eye open. Curr. Biol..

[55] Rattenborg NC, Lima SL, Amlaner CJ (1999). Half-awake to the risk of predation. Nature.

[56] Jolly CJ, Phillips-Conroy JE (2003). Testicular size, mating system, and maturation schedules in wild Anubis and hamadryas baboons. Int. J. Primatol..

[57] Strum SC (1991). Weight and age in wild olive baboons. Am. J. Primatol..

[58] Packer C (1979). Inter-troop transfer and inbreeding avoidance in Papio anubis. Anim. Behav..

[59] R Development Core Team. R: A language and environment for statistical computing, https://www.Rproject.org/ (2017).

[60] Kay, M. & Wobbrock, J. O. ARTool: Aligned Rank Transform, https://cran.rproject.org/web/packages/ARTool/index.html (2016).

[61] Lenth, R. & Love, J. Package ‘Ismeans’, https://cran.r-project.org/web/packages/lsmeans/index.html (2018).

